# Emerging Applications of Positron Emission Tomography in Coronary Artery Disease

**DOI:** 10.3390/jpm15030100

**Published:** 2025-03-03

**Authors:** Anna Blach, Jacek Kwiecinski

**Affiliations:** 1 Department of Cardiology and Structural Heart Diseases, Medical University of Silesia, 40-055 Katowice, Poland; 2 Nuclear Medicine Department, Voxel Diagnostic Center, 40-514 Katowice, Poland

**Keywords:** positron emission tomography, myocardial perfusion imaging, coronary artery disease

## Abstract

Coronary artery disease remains the leading cause of morbidity and mortality worldwide. With the changing clinical manifestation and novel therapeutical options, precise disease phenotyping becomes increasingly important at the point of care. In the management of coronary artery disease, myocardial perfusion imaging (MPI) remains the cornerstone of clinical practice. Although traditionally MPI has been primarily performed with single photon emission computed tomography (SPECT), nowadays, given the changing spectrum of the disease, greater precision and additional assessment of myocardial blood flow are desired. Due to the fundamental advantages of PET over SPECT, i.e., higher spatial resolution, accurate attenuation correction for each scan, and higher count rates, the sensitivity and specificity of PET MPI are higher than those of SPECT MPI and are estimated to be approximately 90–92% vs. 83–88% and 81–87% vs. 70–76%, respectively, according to meta-analysis data. Consequently, over the past decade, we have witnessed an increased uptake of positron emission tomography (PET) MPI. With the improved spatial resolution, the ability to quantify myocardial blood flow, and the potential to depict the burden of coronary atherosclerosis with low-dose computed tomography, PET/CT is uniquely positioned to facilitate a comprehensive non-invasive assessment of disease, providing an opportunity for precision medicine. The wealth of data obtained during a single imaging session can be challenging to integrate at the time of image analysis. There has therefore been an increasing interest in developing predefined thresholds or variables (scores) which combine the multidimensional data acquired with PET MPI. Beyond MPI, PET can also serve for the assessment of disease activity at the atherosclerotic plaque level, further refining our understanding of the biology of coronary artery disease and providing hope for enhanced prediction of myocardial infarctions. In this narrative review, we present the current applications of PET MPI in coronary artery disease and focus specifically on two areas that have recently garnered considerable interest—the integration of multiparametric PET MPI data and coronary plaque activity PET imaging.

## 1. Introduction

The progressive and chronic nature of coronary artery disease necessitates the continuous monitoring of the patient’s condition from the moment of the earliest possible accurate diagnosis until the end of life. Despite the improving allocation of patients for revascularization, including coronary artery bypass grafting (CABG), percutaneous coronary interventions (PCI), or optimal medical treatment, modern therapy does not negate disease progression, which persists as an ongoing threat, ultimately having an adverse impact on patients’ outcomes [1,2].

The non-invasive nuclear cardiology imaging methods positron emission tomography (PET) and single photon emission scintigraphy (SPECT) have recently transformed the management of coronary artery disease. Recording radioactive decay in vivo when gamma-ray photons are released into the patient’s tissues is the common principle behind both modalities. The photons emitted by the radioemitter are detected by the system as distinct SPECT events. PET picture creation is based on the coincidence of photons from the annihilation of positrons emitted from the radiotracer [3]. Nuclear cardiology modalities have been demonstrated to markedly reduce the number of unnecessary invasive procedures [4] and the related costs. Furthermore, published records from both Europe and the United States indicate an upward trajectory in the number of PET MPI examinations conducted over the past decade [4,5].

## 2. Changes in the Patient Population—The Need for a More Personalized Approach

There has been a notable shift in the demographic profile of individuals presenting with coronary artery disease (CAD) in recent years. The proportion of patients with obstructive CAD has decreased as overall we are witnessing an increasing number of patients with diffuse atherosclerosis and associated microvascular dysfunction [6,7,8]. Consequently, while there has been an improvement in the epidemiology of obstructive CAD, the aging population, the increasing prevalence of obesity, and its associated comorbidities [9] have prevailed. This constellation of risk factors has the potential to accelerate the progression of atherosclerosis affecting small vessels and hence leading to a form of disease that can be largely undetectable by complementary non-invasive imaging techniques. In such cases, a detailed perfusion evaluation, such as that conducted using PET, is the gold standard method to elucidate the disease providing a comprehensive response to the vast majority of the posed questions.

## 3. Myocardial Perfusion Imaging

The physiological phenomenon of tissue perfusion, initially defined by August Krogh more than a century ago [10], is utilized in non-invasive cardiac diagnostics employing a range of imaging methods including computed tomography (CT), magnetic resonance imaging (MRI), and nuclear medicine modalities such as PET and SPECT. The latter, which incorporates visual and semi-quantitative evaluations of perfusion, has been the most prevalent modality in clinical practice for several decades, primarily due to the extensive accessibility and cost-effectiveness of the procedure. However, some of the main limitations of conventional SPECT MPI include its low sensitivity compared to PET MPI in detecting multivessel coronary artery disease, left main stenosis, and microvascular dysfunction [10,11]. Conversely, PET MPI, despite its numerous advantages over SPECT perfusion assessment, has been utilized less frequently due to its more stringent technical requirements, cost implications, and the limited availability of skilled professionals [12,13].

PET imaging involves comparing myocardial perfusion at two points: at rest and at the highest myocardial oxygen demand obtained during the stress test, i.e., the assessment of myocardial flow reserve (MFR). MFR defines the maximum possible increase in myocardial blood flow (MBF), expressed as a percentage of baseline, and hyperemic flow. In healthy individuals, the MFR is 300–600%, meaning that when the heart’s oxygen demand is maximal, it increases 3–6 times without disrupting myocardial perfusion. A quantitative measurement that provides numerical mathematical data on myocardial blood flow at rest and stress, which can be evaluated independently in all myocardial segments, is therefore the best way to assess the baseline/rest and induced/stress flow and MFR in detail. Numerous studies have shown that, regardless of the method of assessment, reduced MFR is associated with an increased risk of death and major adverse cardiovascular events (MACEs) [14]. The advent of new technologies, including high-speed SPECT cardiac cameras, has made it possible to obtain coronary flow parameters using this method; however, although promising, the results require further study. The data regarding the sensitivity and specificity of PET and SPECT are inconsistent, as evidenced by various meta-analyses. This inconsistency is attributable to the utilization of disparate detectors and tracers in the studies, as well as the observation of diverse populations exhibiting varying degrees of coronary heart disease (CHD) progression. It is generally acknowledged that sensitivity and specificity for PET and SPECT are 90–92% vs. 83–88% and 81–87% vs. 70–76% [15].

## 4. CZT-SPECT, MRI, and CT Perfusion

Recent years have also witnessed the emergence of alternative methods for assessing myocardial perfusion, including CZT-SPECT (cadmium-zinc telluride single photon emission tomography), MRI, and CT.

**Figure 1:**
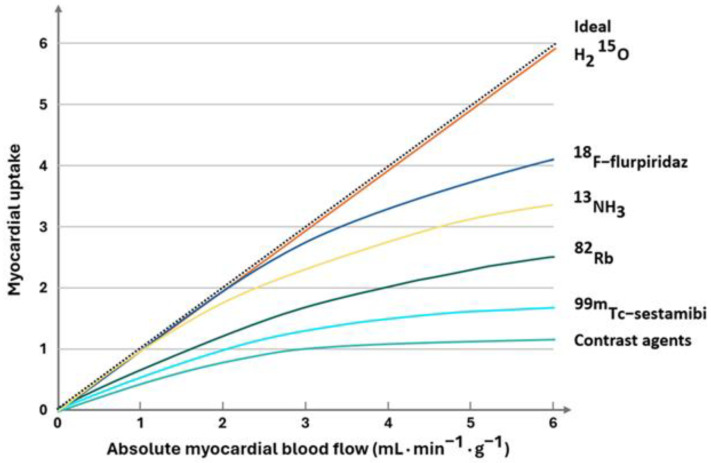
Radiotracer first-pass extraction. Estimates of the relationship between myocardial uptake and myocardial blood flow for radiotracers and contrast agents.

The availability of fast cardiac CZT solid-state cameras has enabled the dynamic assessment of MFR and MBF using this method. And although the characteristics of the radiotracers (Figure 1) and the resolution, which are somewhat inferior to PET, are obvious limitations, the easier availability, lower costs, and convenient access to technetium-labelled radiotracers have led to a significant increase in interest in dynamic SPECT MPI. The dynamic examination protocol consists of several phases, similar to PET-MPI, but does not include computed tomography, as heart-focused CZT devices that are not hybrid scanners are not routinely equipped with CT system. Since the Wells study in 2014, which showed that it is possible to evaluate dynamic blood flow and absolute coronary flow reserve in a porcine model [16], as in good PET, it has become possible to evaluate MBF and MFR. The first comparative studies, such as the Waterday study, showed a similar MFR compared to PET using ^15^O-water and a high diagnostic value for detecting impaired MFR and abnormal FFR in conventional angiography in patients with stable CAD [17]. The cut-off value for MFR was assumed to be 2.1, transferring this number from the evaluation in PET. Indeed, it is known that the MFR to FFR reference is not entirely correct because FFR does not show microvascular impairment. Therefore, it is more important to compare CZT SPECT to MFR in PET, as demonstrated in many studies [18,19,20]. The evaluation of perfusion in MRI has been demonstrated to be a well-validated modality [21]. In comparison to CT, MRI has been shown to be more accurate and reliable in detecting myocardial ischemia, yet due to the long acquisition time, the partial volume effect, and common motion artifacts, subendocardial ischemia evaluation remains challenging. However, further research is necessary to fully explore the potential of CT perfusion. The use of dual-source scanners has led to the generation of promising data, suggesting the potential for future applications of this method [22,23]. In both cases, however, we are dealing with perfusion assessment with a contrast agent serving as a “tracer” that is far from the linear kinetics of PET tracers (Figure 1). The fundamental characteristics of these three modalities are summarized in Table 1.

## 5. Principles of PET Absolute Myocardial Blood Flow Assessment

To quantify coronary flow, the patient is injected intravenously with one of the perfusion tracers (Table 2) at two points in the study: at baseline and at the peak pharmacological stress-induced hyperemia. In a procedure where both steps are performed in sequence, the PET-CT scanner performs a dynamic image acquisition, which commences immediately before the tracer is injected intravenously. The calculation of MBF is enabled by the tracking the tracer in the bloodstream and subsequently in the myocardium, through the utilization of appropriate mathematical kinetic modeling based on image-derived time-activity curves. For the most commonly used tracers, ^82^Rubidium and ^13^N-ammonia, two models are utilized: the 1-tissue compartment model [24,25,26] and the simplified retention model [27,28]. Alternative approaches for quantifying MFR have been recently proposed. Juneau et al. proposed an additional method for assessing MFR using only static perfusion images. This approach could be widely applicable to older-generation 3D PET and PET-CT scanners, employing a simplified method to estimate MFR based on the stress–rest ratio in patients referred for routine ^13^N-ammonia or ^82^Rubidium MPI PET [29,30]. Another simplified quantification of MFR with Flurpiridaz is based on the rest-stress Standardized Uptake Value measurement [31]. Although promising, both the latter approaches require further evaluation and blinded validation.

## 6. Radiotracer Characteristics

The characteristics of the radiotracer are directly related to the accuracy of the test. The greater the tracer uptake with increasing coronary flow, the more accurate the assessment of the MFR and the ability to see changes in flow. In other words, the greater the linearity of uptake, the more sensitive the test. Hence, oxygen-15-labeled water (^15^O-water) is an ideal tracer, as it diffuses inertly into and out of the myocyte and shows a linear 1:1 correlation between increasing blood flow and “uptake” ratio in the myocardium (Figure 1) [32]. ^15^O-water PET MPI serves thus as a reference standard for other modalities evaluating myocardial perfusion including SPECT [17], MRI [33], computed tomography fractional flow reserve (FFR) [34], and invasive angiography [35]. Despite the preferential pharmacokinetics, ^15^O-water has a short half-life and therefore alternative tracers have a greater share of the market.

^82^Rubidium, a potassium analogue, needs energy for myocardial uptake through Na/K-ATPase. Its significantly flatter uptake curve (Figure 1) but high retention rate and relatively convenient accessibility compared to cyclotron tracers make it the most widely used tracer in the world [36].

While ^13^NH_3_ offers good imaging parameters due to its 10 min half-life similar to ^15^O-water, it mandates an onsite cyclotron, which limits its applicability. Following its uptake, ammonia cell trapping and conversion to glutamine permit the generation of scans of quality. ^18^F Flurpiridaz—a relatively new radiotracer on the market, is widely considered as an optimal MPI agent to date: labeled with fluorine-18 with it, it has the second-best flow characteristics after ^15^O-water and a superior upper range of flow compared to the remaining available tracers (Figure 1). As the recent multicenter AURORA trial showed, ^18^F Flurpiridaz with its excellent image quality is superior to SPECT for CAD detection, specifically in patients who are more difficult to image such as women and obese individuals [37] or subjects with smaller left ventricles [38]. Alternative tracers labeled with ^18^F with longer half-lives are undergoing clinical trials and may soon further broaden our armamentarium [39,40,41,42,43].

## 7. Myocardial Flow Reserve

**Figure 2:**
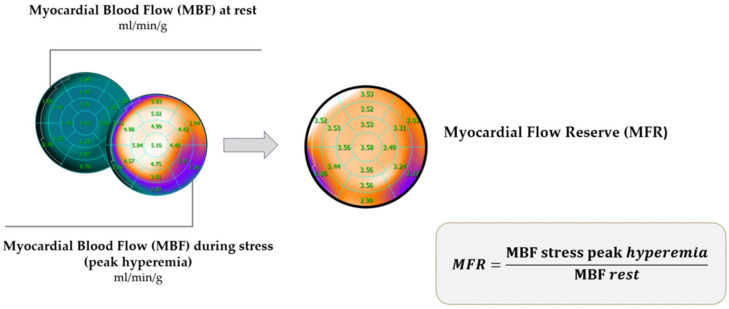
Myocardial flow reserve (MFR) calculation.

Myocardial flow reserve (MFR), a noninvasive analogue to coronary flow reserve, is a physiological index for the identification of myocardial flow impairment. The assessment of MFR is a requisite component of each dynamic PET MPI and does not necessitate the acquisition of additional data or an increase in the administered radiotracer dose. MFR reflects the disease burden in the entire coronary vasculature, including epicardial coronary artery stenosis, diffuse atherosclerosis, and microvascular function. It is defined as the ratio of the peak stress hyperemic myocardial blood flow (MBF) to the resting myocardial blood flow (Figure 2).

However, this is contingent upon the stressor employed and the nuclide utilized in the study [44]. A summary of the PET tracers that are currently available for use in conventional myocardial perfusion imaging and quantitative microvascular blood flow imaging can be found in Table 2. It should be borne in mind that, in addition to the technical parameters, MFR is influenced by diverse non-mathematical factors, e.g., age, sex and BMI [45]. These variables were taken into account in establishing the standard for this parameter. Currently, the normal MFR is considered to be >2; values < 2 are considered pathological. MFR has important diagnostic and prognostic implications in evaluating and managing patients with known or suspected CAD.

## 8. MBF at Rest and Stress

Resting MBF in healthy individuals ranges from 0.4–1.2 mL/min/g and depends on oxygen demand, which correlates with the rate-pressure product (RPP, defined as the product of systolic pressure and heart rate) [46]. Its values are higher in older age, individuals with high degree of obesity, and in women or due to the holding of beta-adrenolytics/antihypertensive drugs on the day of study [47]. Indeed, increased resting MBF affects the value of the calculated MFR despite normal/almost-normal peak stress MBF, and correction should then be implied to avoid the misassessment of the MFR and an increased risk of MACEs. However, as demonstrated by Huck et al., RPP correction may not be applicable in patients without obstructive CAD. In patients with normal perfusion and an MFR < 2.0, corrected MFR derived from corrected rest MBF values might not consistently offer adequate risk categorization [48].

Stress MBF reflects the maximal myocardial blood flow during pharmacologically induced hyperemia after the administration of an inotropic agent or vasodilators. Peak stress hyperemic flow values regarded as normal are usually >1.7 mL/min/g. Stress MBF is a parameter that needs to be considered when evaluating MFR. The clinical data show that the impairment of both peak stress MBF and MFR are independent risk factors for MACE. Indeed, when analyzed in an integrated manner, they permit the reclassification of risks and prognosis [49,50]. Moderate reductions in stress MBF or MFR are often due to the effects of diffuse CAD and microvascular dysfunction [51]. Determining the precise normal hyperemic MBF value requires careful consideration, as the term “hyperemic MBF value” is not straightforward. The adoption of different cutoff values as normal, physiological values in clinical trials is due to variations in research methods, kinetics of the tracers, and the equipment used. These variations are illustrated in Table 3 [52,53,54,55,56,57,58,59,60]. The blood flow and MFR values can refer to either the entire myocardium or to a specific area, such as a vascular territory, wall, or a single segment. When analyzing these parameters in patients with CAD, e.g., after a myocardial infarction, this is of great relevance. In such patients, the actual measurement of real-time hyperemic MBF will be more important than MFR. In the area of a post-infarction scar, resting blood flow is usually significantly reduced, as is hyperemic MBF, which give low MFR. In the case of non-transmural scar, the hyperemic MBF will oscillate at slightly higher values: substituting the data into the mathematical formula (Figure 2) will result in a falsely high MFR.

**Figure 3:**
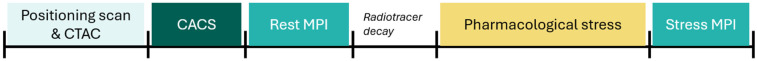
Schematic representation of the multi-parametric PET-CT protocol using perfusion radiotracers: ^13^N-ammonia or ^82^Rubidium.

The schematic below (Figure 3) shows a typical dynamic PET MPI protocol. Current hybrid PET/CT scanners routinely acquire CT data to correct for PET data attenuation. Contrast-enhanced coronary CT angiography (CCTA) can also be performed in a single imaging session to assess coronary anatomy. However, this is not a routine procedure. It requires the administration of an additional contrast agent and an additional dose of radiation.

## 9. Guidelines

The indications for PET MPI in patients with CAD have been outlined in the latest 2024 ESC clinical practice guidelines for the management of patients with chronic coronary syndromes. European recommendations include the use of PET MPI in two clinical scenarios:

(a) In the initial diagnostic management of individuals with suspected disease and moderate or high (>15–85%) pre-test likelihood of obstructive CAD to diagnose and quantify myocardial ischemia and/or scar, estimate the risk of MACEs, and quantify myocardial blood flow. The guidelines point out that it is of vital importance to assess myocardial perfusion using PET-CT, particularly in obese patients (high photon energy), in young patients (low radiation dose exposure), and in those with known or suspected diffusely impaired MBF, e.g., those with multivessel CAD or microvascular dysfunction.
(b) In patients with known CAD for the identification of an increased risk of MACEs demonstrated by PET MPI in an area of ischemia ≥ 10% of the LV myocardium (class I, level of evidence B).

## 10. Usefulness of Quantitative PET Functional Assessment

What we have been facing over recent years are evolving paradigms for revascularization strategies in CAD from anatomy-driven data to the current combination of anatomy and functional myocardial flow data. It can be posited that the utilization of algorithms to qualify patients for revascularization based exclusively on anatomical imaging is a relic of a bygone era. In the domain of invasive diagnostics, there is a necessity for functional evaluation, such as the measurement of fractional flow reserve (FFR), as opposed to making therapeutic decisions based solely on the visual evaluation of angiography.

In patients with obstructive coronary artery disease (CAD), the optimal strategy for improving survival remains a topic of ongoing research and debate. To date, there are few randomized controlled trials evaluating the effect of percutaneous coronary interventions (PCI) on mortality compared with those who received conservative treatment [61,62,63,64]. However, there is suggestive evidence that if patients eligible for PCI were selected on the basis of an imaging parameter such as MFR, the benefits would be apparent [65,66]. In a study by KK Patel et al. conducted between 2010 and 2016 in 12,594 patients undergoing PET MPI, a reduction in MFR was associated with an elevated risk of all-cause death; patients with an MFR ≤ 1.8 had a survival benefit with early revascularization, regardless of the type of revascularization or magnitude of ischemia [67]. These initial results were confirmed in a recent multicenter publication involving patients with diagnosed CAD and induced ischemia of ≥5% eligible for revascularization. Toftholm et al. demonstrated that an MFR < 2 can be used to identify those with a better prognosis and benefit from revascularization [53]. The integration of hyperemic myocardial blood flow and coronary flow reserve, coronary flow capacity (CFC), is another detailed measure outperforming other perfusion metrics to clarify functional significance [41,42,43].

The reduction in MFR has been identified as a robust prognostic indicator in a well-studied cohort of patients with heart failure. In these patients, the viability assessment alone would appear insufficient to determine eligibility for revascularization [65,68,69]. An interesting approach to the assessment of CFC, the observed and hypothetical impact of this parameter on mortality and outcome after revascularization, was presented in a series of papers by Gould et al. [70]. These data highlight that reduced CFC and global MFR on PET MPI directly translate into an increased risk of MACEs, and that revascularization, particularly CABG, also has a measurable benefit in improving these parameters [70,71,72]. Beyond atherosclerotic coronary disease, heart transplant patients can also benefit from PET MPI. In this population, multiparametric PET is demonstrating considerable efficacy in identifying microvascular dysfunction, which is a hallmark of cardiac allograft vasculopathy. Reduced MFR is associated with a higher risk of the composite outcome of mortality, transplantation, heart failure hospitalization, acute coronary syndrome, or revascularization [65,73,74,75,76]. As demonstrated in recent years, the quantitative assessment of perfusion in PET is becoming increasingly useful in both diagnostics and risk assessment, as well as prognosis. The correlation with invasive indicators, such as FFR, facilitates a problem-free evaluation of the significance and severity of the disease at the time of diagnosis. In comparison to other non-invasive imaging methods, under real-world conditions, a smaller number of patients require further tests after PET MPI to reach a conclusive diagnosis [77]. The ISCHEMIA study showed that imaging tests alone, demonstrating the severity of ischemia, is not sufficient to determine the benefit of revascularization [64]. However, it appears that the inclusion of quantitative flow parameters will allow the identification of patients suitable for revascularization versus conventional therapy. This would allow therapeutic decisions to be based to a large extent on quantitative PET data alone. The intervention arm of the CENTURY trial was the first randomized trial to serially assess quantitative PET-MPI at baseline and 2 and 5 years to guide revascularization decisions [70]. Another randomized study, PERFORM-CCS, is ongoing and plans to enroll 570 patients. Such large-scale trials with multiple-patient participation ensure the standardization of data collection through the implementation of unified examination protocols and reporting within a designated medical facility. In the future, there is a necessity to establish multicenter data collection initiatives. Presently, this is feasible and progressively more straightforward due to the integration of artificial intelligence, which facilitates the development and interpretation of images.

Diagnostic and prognostic data derived from a normal or impaired MFR are presented in Table 4.

## 11. Multiparametric Approach: CT

While the characterizations of myocardial perfusion and blood flow are the key features of PET MPI, left ventricular function and coronary calcification (CAC) can further improve the diagnostic accuracy of PET. Indeed, in clinical practice, reading physicians combine this information to distinguish patients who should proceed to invasive coronary angiography [78]. Initial efforts to incorporate CAC into multiparametric models have shown encouraging results. Zampella et al. demonstrated that combining CAC, MBF, and MFR provides incremental information about the presence of CAD [79]. Similarly, Brodov et al. [80] showed that integrating per-vessel ischemic TPD with CAC improves CAD detection. Historically, studies combining PET MPI with CAC data were hampered by the availability of CAC information. Indeed, traditionally, disease burden assessments on non-contrast CT have been obtained exclusively on dedicated ECG-gated CT scans [81]. More recently, it was demonstrated that CAC can be quantified from low-dose ungated CT attenuation correction maps which are routinely acquired during PET/MPI.

**Figure 4:**
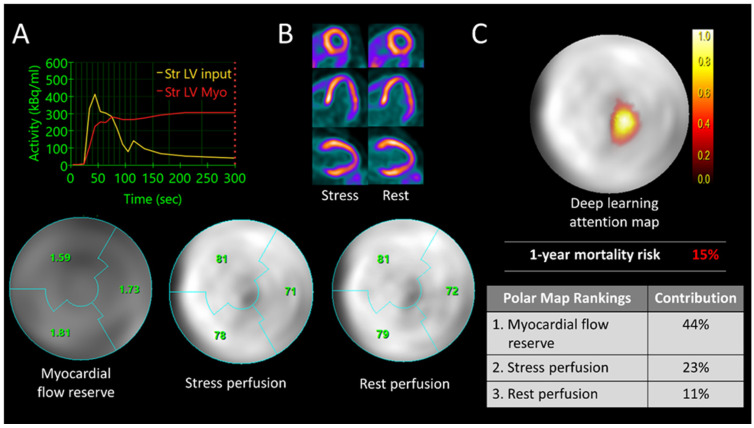
Case example of mortality risk assessment with the use of deep learning in multiparametric PET. (**A**) A 72-year-old female patient was observed to have a reduced coronary flow reserve of 1.7, as indicated by the polar map. (**B**) Her rest and stress perfusion were found to be normal. The patient passed away three months following the PET scan. Image (**C**) shows the deep learning attention map: patient’s individual risk of mortality. Adapted with permission [83]. LV: left ventricle, Myo: myocardium.

In a large multicenter cohort, Pieszko et al. [82] demonstrated that CAC can be derived fully automatically and rapidly with a deep learning approach. Importantly, in comparison to standard CAC scoring, the AI-based assessments performed using CT attenuation correction scans predicted major adverse cardiovascular events equally well. Indeed, the negative predictive value of deep learning scores did not differ significantly, and standard CAC assessments did not provide significant reclassification improvement over the fully automatic approach. These recent developments provide hope that in the future, the full extent of PET-/CT-derived measurements shall be fully harnessed for optimal diagnostic performance. Ideally, the full integration of this wealth of data could be facilitated by artificial intelligence approaches whose role is rapidly increasing in nuclear cardiology (Figure 4). 

## 12. Coronary Plaque Imaging

Beyond imaging myocardial perfusion and flow, PET can also serve for direct assessments of disease activity in the coronary vasculature. Initially, tracer uptake was observed in ^18^F-Fluorodeoxyglucose (FDG) PET imaging. Despite the pivotal role of inflammation in atherosclerotic plaque formation, progression and rupture ^18^F-FDG PET turned out to be suboptimal due to the pronounced myocardial uptake which results in significant overspill and hence hampers identification ^18^F-FDG activity within coronary plaques. While this limitation can be overcome with dietary preparation, to date, we lack outcome data supporting the wider use of ^18^F-FDG coronary PET [84,85]. Indeed, observational studies have largely focused on establishing the feasibility of ^18^F-FDG coronary imaging. Recently alternative PET tracers depicting the inflammatory response within atherosclerotic lesions have been proposed. Both ^68^Ga-Pentixafor [86,87,88,89] and ^68^Ga-Dotate [90] have shown hope, yet again, prognostic implications remain to be addressed in future studies.

## 13. Metabolic Activity and Microcalcifications Visualization: ^18^F NaF

While inflammation plays a major role in atherosclerosis, calcification processes are a hallmark of vascular pathology [91]. Indeed, in clinical practice, assessments of the burden of calcified lesions are used to guide management [81]. Importantly, aside from serving as an indicator of the presence of disease, developing calcifications has been identified as markers of progressing and potentially vulnerable plaques [92]. Indeed, microcalcifications are considered adverse plaque features, and pathological studies have suggested that their presence might contribute to lesion rupture triggering an acute event [93]. In view of the pivotal role of calcification in atherosclerosis, there has been increasing interest in imaging the activity of the calcification process. Luckily, by means of ^18^F-sodium fluoride, it is now possible to characterize these processes non-invasively [94]. Initially utilized as a bone tracer, ^18^F-NaF uptake has been widely reported on whole body imaging within atherosclerotic plaques [95]. On whole-body PET, the activity of ^18^F-NaF has been linked to unfavorable risk profiles, the extent of the disease, and the symptomatic status [96]. Subsequently, dedicated ^18^F-NaF coronary imaging commenced. By employing longer emission scanning, ECG-gating, despite the relatively microscopic target ^18^F-NaF PET, has enabled quantifying the activity of atherosclerotic plaques on the individual lesion, vessel, and patient level [97,98]. In a landmark observational study of patients with a recent type 1 myocardial infarction, Joshi et al. established that increased ^18^F-NaF is a universal finding within culprit plaques [99]. This observation has fueled further studies which explored the interplay between plaque morphology and activity as well as sought to test the prognostic implication of ^18^F-NaF coronary uptake.

**Figure 5:**
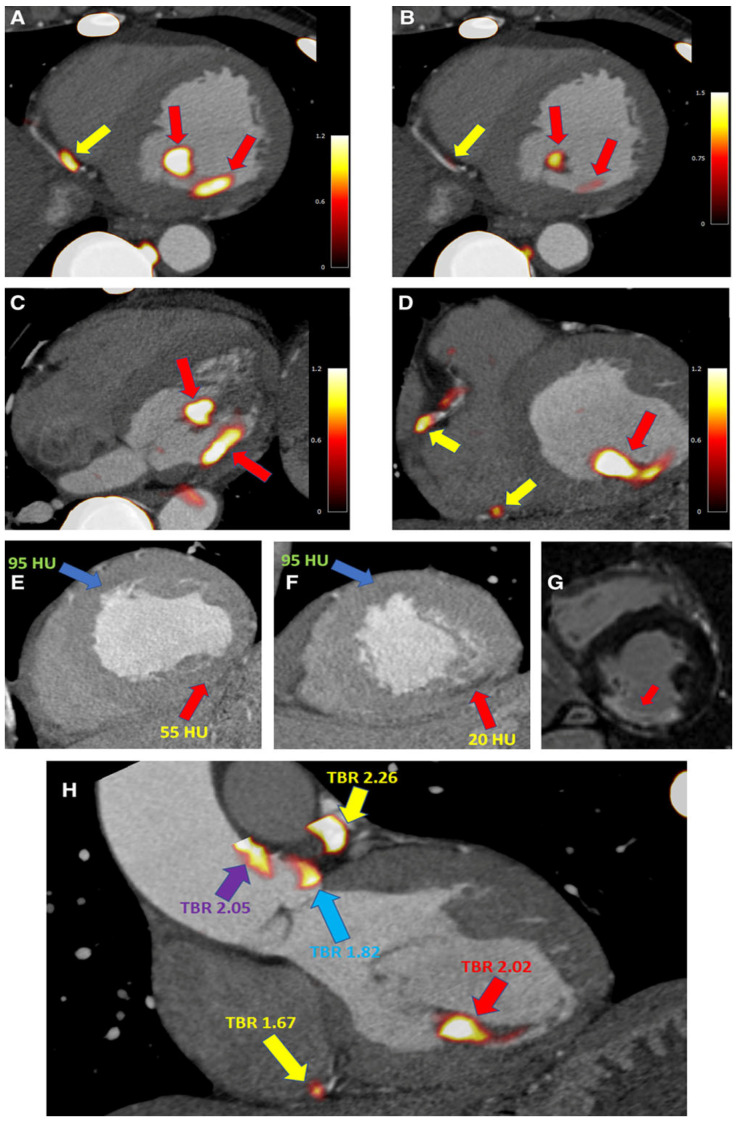
A 67-year-old patient’s Na-F PET-CT scan: (**A**) PET-CT axial plane shows three foci of radiotracer uptake: two in the inferomedial papillary muscle (red arrows) and one in a distal RCA (yellow arrow). (**B**–**D**) Comparing the intensity of the uptake, a higher absorption and increased activity of radiotracer in papillary muscle (red arrows) than in coronary artery (yellow arrows). (**E**,**F**) Computed tomography angiography (CTA) scans utilized for anatomic reference: blue arrows indicate an area of low attenuation located in the anteroseptal wall; red arrows show inferior left ventricular (LV) wall and posteromedial papillary muscle (red arrows). Similar patterns of attenuation are seen in myocardium after the infarction, with foci of microcalcification development (which accumulates ^18^F-NaF). If CTA is not available for precise anatomical localization, the foci of intense ^18^F-NaF uptake may be mistakenly attributed to atherosclerotic plaques in the posterior left ventricular artery or the posterior descending artery. (**G**) MRI scan showed regions of delayed enhancement (white) equal to the scarring of the inferior wall and posteromedial papillary muscle. (**H**) Another localization of uncommon extracoronary 18-F NaF uptake in the aorta, in the absence of CTA, may be assigned to one of the proximal left coronary artery plaques (purple arrow); coronary atherosclerotic plaques in RCA and LAD (yellow arrows); infarcted papillary muscle (red arrow); Uptake next to the left coronary cusp of the aortic valve (blue arrow). LAD—left anterior descending artery; RCA—right coronary artery. Adapted with permission [119].

The growing number of ^18^F-NaF coronary papers has coincided with the expanding application of coronary CT angiography [100]. The latter techniques enable in vivo assessments of coronary plaque morphology and composition [101]. Recent studies have suggested that lesions with adverse morphology and in particular low attenuation plaque, consistent with a necrotic core, serve as a biomarker of adverse prognosis [102,103]. Interestingly, multimodality studies have demonstrated that adverse plaque morphology is linked to ^18^F-NaF uptake, suggesting that plaque activity and morphology are associated with one another [104]. These observations have been consistently reported across the spectrum of coronary atherosclerosis ranging from suspected to established disease [99]. Likewise, more advanced methods for CT-angiography-based plaque phenotyping, such as radiomics, highlighted that plaque activity and morphology are associated [105,106]. Similar findings were reported in analyses employing intravascular ultrasonography imaging with adverse morphology showing strong correlations with ^18^F-NaF uptake [107]. Longitudinal studies with baseline ^18^F-NaF PET/CT angiography and follow-up CT have shown that PET tracer uptake predicts disease progression. Indeed, this observation was a universal finding across vascular beds, including native coronary arteries, bypass grafts, and great vessels [108,109,110,111]. Given that similar associations have been shown in the realm of valvular heart disease, including native aortic stenosis and aortic valve bioprosthesis, ^18^F-NaF uptake in PET is now widely considered as a robust predictor of disease progression across a wide range of cardiovascular conditions (Figure 5) [112,113,114].

The encouraging findings of the aforementioned mechanistic studies have led to observational outcome analyses. These were initially retrospective post hoc analyses of multicenter data. In a study encompassing 293 patients with established multivessel disease who were followed-up with for over 4 years, it was demonstrated that increased ^18^F-NaF uptake is a strong independent predictor of myocardial infarction [115]. Indeed, while patients with no tracer uptake had an excellent prognosis, a third of the population with a coronary and a high microcalcification activity (which is a measure that considers both the extent and intensity of uptake) had an 8-fold increase in the risk of acute coronary events. These promising findings have been confirmed in an enlarged cohort involving patients with concomitant valvular disease, and it has also been shown that further improvement in the accuracy of prediction of myocardial infarction can be achieved by combining PET data with detailed coronary CT-angiography-based plaque assessments [116]. Recently, in the PREFFIR study, which recruited over 700 patients with a type 1 myocardial infarction who underwent ^18^F-NaF PET within 3 weeks of the acute event, ^18^F-NaF uptake emerged as an independent predictor of the combined endpoint encompassing myocardial infarction and cardiac death [117]. Despite the underrepresentation of women in the majority of ^18^F-NaF coronary PET studies, a recent analysis confirmed that this imaging test performs equally well in men and women, consistently predicting myocardial infarction or cardiac death irrespective of sex [118].

## 14. A New Era—Artificial Intelligence in PET Imaging

Positron emission tomography in coronary artery disease has recently benefited from technological progress. New scanner designs with digital photon counting have been shown to improve image quality and hence can potentially further enhance the clinical utility of the test [120,121]. Multiparametric imaging has one major drawback: voluminous data that need to be analyzed. Machine learning is a process that utilizes a substantial volume of data, employing algorithms that are designed to identify correlations and patterns. These patterns facilitate the formulation of conclusions and predictions through a process of unsupervised learning and refinement. This is where AI has come in over the last decade: starting with basic parameters, deep-learning-based image registration and motion correction make it possible to further improve image quality, shorten examination times, and avoid artifacts that negatively affect the interpretation of flow values [122]. The effectiveness and precision of MBF quantification have been improved by using artificial intelligence to directly predict parameters from pharmacokinetic models [123]. The next area of its implementation tends to be cardiovascular risk prediction: combining clinical and MPI data, AI is highly accurate in MACE risk assessment as Betancur et al. showed [124]. Considering the accompanying progress in image protocols and a greater uptake in motion correction techniques, PET will likely experience an exponential growth in the coming years [125,126,127,128]. In particular, with the advent of artificial intelligence approaches which have already been shown to greatly facilitate combing the wealth of information provided by PET, we might witness further dissemination of PET beyond academic centers [129]. But before fully incorporating AI into clinical real-world practice, first of all, we have to create multi-institutional partnership and standardize algorithms. Regulatory agencies ought to help with legal and procedural regulations and ongoing monitoring for secure clinical use.

Given the growing availability of digital PET/CT scanners, the increasing role of artificial intelligence, and the constant refinement of imaging protocols, in the future, we shall likely witness further progress in the diagnostic and prognostic accuracy of PET imaging in coronary artery disease.

## 15. Limitations of PET Imaging Technique in Coronary Artery Disease

The primary constraint imposed by PET technology pertains to its limited accessibility. The tracers utilized in PET possess a short half-life, necessitating their synthesis within an onsite cyclotron at the facility. An alternative option involves the use of ^82^Rb which is produced in a generator. However, both the cost of ^82^Rb and that of cyclotron tracers are notably high, impeding their widespread utilization. Consequently, PET scans are employed in a restricted manner, reserved for cases that are typically challenging to diagnose. A significant challenge in multiparametric assessment arises from the absence of a standard CT scan with contrast, which hinders the correlation of perfusion abnormalities detected by PET with the corresponding coronary vessels, only discernible after contrast administration. The procedure entails the use of ionizing radiation, a potential hazard to the patient. Radiation exposure originates from an isotope administered intravenously and an external CT scanner. The interpretation of such an examination is challenging and necessitates skilled, experienced professionals. Perfusion testing with dynamic assessment (flow parameters) poses significant difficulties in patients with advanced CHD, particularly following a myocardial infarction and/or a revascularization procedure.

## 16. Conclusions

With the changing clinical profile of patients with coronary artery disease, non-invasive diagnostic imaging is applying increasingly sophisticated techniques and individual patient data to more accurately assess blood flow and ischemia. This approach improves diagnostic accuracy, personalizes treatment strategies, and predicts outcomes by taking into account patient-specific anatomical and physiological variations that are often overlooked by complementary methods. Despite the growing number of studies and available evidence, further assessment is needed to standardize cut-off values for individual PET parameters, in particular for PET-guided revascularization procedures.

## Figures and Tables

**Table 1:** Myocardial perfusion imaging techniques and their fundamental features. (*) Coronary artery assessment is not routinely implied, but available as coronary calcium scoring or when a contrast agent is injected as contrast computed tomography. (+) low, (++) moderate, (+++) high, (++++) excellent.

	PET	SPECT	MRI	CT
Basic application	functional: perfusion and metabolism	functional: perfusion and metabolism	functional and tissue characterization	detailed anatomical imaging
Radiation	low	high/moderate	no	high/moderate
Contrast agent	no	no	yes	yes
Aquisition time	short	long	long	short
Temporal resolution	++	+	+++	++++
Spatial resolution	++	+	+++	++++
Kinetic model validation	excellent	not available in conventional SPECT. In CZT SPECT under evaluation	good	under evaluation
Coronary anatomy assessment	No *	no	partially possible	excellent
Impaired renal function	does not negatively affect kidney function	does not negatively affect kidney function	contraindicated if severe: eGFR < 30 mL/min/1.73 m^2^	caution if eGFR < 59 mL/min/1.73 m^2^, contraindicated if severe: eGFR < 30 mL/min/1.73 m^2^
Ventricle function assessment	moderate	moderate	excellent	good
Availability	low	moderate	moderate/low	high
Costs	high	moderate	moderate	moderate/low

**Table 2:** Positron Emission Tomography tracers used for myocardial perfusion imaging.

Radiotracer	Source	Half-Life (min)	Max Rest/Stress Extraction (%)	Max Rest/Stress Retention (%)	Extracardiac Uptake	Effective Dose (mSv/GBq)
^15^O-water	cyclotron	2.0	~100	No retention	Liver	1
^82^Rb-chloride	generator	1.27	~70/35	70/25	Stomach	1
^13^N-ammonia	cyclotron	10	100/95	90/50	Liver and lungs	2
^18^F-flurpiridaz	cyclotron	110	~100/95	90/55	Liver	20
^18^F-XTR004 ^18^F-HX01 ^18^F-SYN2	cyclotron	110	Clinical trials			

**Table 3:** A selection of examples of research findings as well as MFR/MBF PET cut-off values. MFR—myocardial flow reserve; MBF—myocardial blood flow; FFR—fractional flow reserve; DS—diameter stenosis.

	Main Aim	Pts Number	PET MPI Tracer	Cut-Off Values for MFR and Hyperemic MBF (mL/min/g)	Significant Coronary Stenosis Criteria	Comments
Danad et al., 2014 [57]	Evaluation for CAD, lesion severity measured with FFR, and its influence on subendocardial perfusion.	66	[^15^O]H_2_O, adenosine	MBF 2.20	FFR ≤ 0.80	Transmural perfusion gradient is impaired in ischemic myocardium.
Danad et al., 2014 [56]	Evaluation for CAD, PET diagnostic accuracy, and cut-off values for MFR and MBF determination.	330	[^15^O]H_2_O, adenosine	MBF 2.3 and MFR 2.5	DS > 90% and/or FFR ≤ 0.80	High quantitative MBF accuracy, focus on sex and age influence on flow parameters.
Joutsiniemi et al., 2014 [55]	MFR and sMBF in detecting significant CAD.	104	[^15^O]H_2_O, adenosine	MBF 2.4, MFR < 2.5	DS > 50% and/or FFR ≤ 0.80	Absolute stress MBF is superior to MFR in detecting significant CAD.
Stuijfzand et al., 2015 [54]	Confirmed CAD patients, non-invasive determination of FFR called relative flow reserve (RFR).	92	[^15^O]H_2_O, adenosine	MBF 2.35, PET CFR 2.58	DS > 90% and/or FFR ≤ 0.80	Non-invasive determination of FFR is possible, no evident improvement of diagnosis accuracy.
Koenders et al., 2022 [59]	Patients suspected of CHD	1519	^82^Rb, regadenoson	MBF ≤ 2, MFR < 2.5	DS 70% stenosis in the LAD, LCX or RCA, or >50% LM	Diagnostic value of segmental MFR is higher than global MFR.
Al Rifai et al., et al. 2023 [60]	CAD patients with prior CABG	836	^82^Rb, adenosine or regadenoson	MFR < 2		Higher risk of MACEs in patients with MFR < 2.0 after CABG.
Højstrup et al., 2023 [58]	Symptomatic patients suspected of CAD; registry-based study	7169	^82^Rb, adenosine or regadenoson	MFR ≤ 2		High predictive value of MFR ≤ 2.0 in all-cause mortality
Toftholm et al. 2024 [53]	CAD patients with perfusion defects were revascularized.	1806	^82^Rb, adenosine or regadenoson	MFR < 2		Better prognosis in patients with low MFR
Winther et al., 2024 [52]	Patients suspected of CHD	Approx. 1000	[^15^O]H_2_O, ^82^Rb	For ^82^Rb MBF ≤ 2.3, for [^15^O]H_2_O MBF < 2.0	DS > 90% and/or FFR ≤ 0.80	Ongoing

**Table 4:** Myocardial flow reserve on positron emission tomography: prognostic and diagnostic data in relation to normal or impaired MFR.

	Normal MFR	Impaired MFR
Diagnosis	Exclude high-risk obstructive CAD when combined with normal perfusion Exclude microvascular dysfunction Exclude cardiac allograft vasculopathy	Confirms CAD, 1-, 2- or 3-vessel disease if abnormal in 1-, 2- or 3-vessel territory Identifies balanced ischemia Identifies >50% left main disease Identifies microvascular dysfunction Frequently seen after ischemic episodes, e.g., myocardial infarction, CABG, PCI. Useful when compared with previous results if available. Identifies no-responder to vasodilator
Prognosis	Very high negative predictive value	MFR 1.7–2: relative risk intermediate MFR 1.2–1.7: relative risk high MFR <1.2 with a perfusion defect: relative risk very high

## Data Availability

No new data were created or analyzed in this study.

## Abbreviations

The following abbreviations are used in this manuscript:

BMI	Mody Mass Index
CABG	Coronary Artery Bypass Grafting
CAC	Coronary Artery Calcium
CAD	Coronary Artery Disease
CCTA	Contrast-enhanced Coronary CT angiography
CFC	Coronary Flow Capacity
CT	Computed Tomography
CZT-SPECT	cadmium-zinc telluride single photon emission tomography
ECG	Electrocardiogram
FDG	Fluorodeoxyglucose
FFR	Fractional Flow Reserve
MACEs	Major Adverse Cardiovascular Events
MBF	Myocardial Blood Flow
MFR	Myocardial Flow Reserve
MPI	Myocardial Perfusion Imaging
NaF	Sodium Fluoride
PCI	Percutaneous Coronary Interventions
PET	Positron Emission Tomography
RPP	Rate Pressure Product
SPECT	Single Photon Emission Tomography
TPD	Total Perfusion Deficit
